# Reduced likelihood of the Poggendorff illusion in cerebellar strokes: a clinical and neuroimaging study

**DOI:** 10.1093/braincomms/fcad053

**Published:** 2023-03-06

**Authors:** Yuichi Higashiyama, Miho Kuroki, Yosuke Kudo, Tomoya Hamada, Keisuke Morihara, Asami Saito, Yosuke Miyaji, Katsuo Kimura, Hideto Joki, Hitaru Kishida, Hiroshi Doi, Naohisa Ueda, Hideyuki Takeuchi, Ken Johkura, Fumiaki Tanaka

**Affiliations:** Department of Neurology and Stroke Medicine, Graduate School of Medicine, Yokohama City University, Yokohama 236-0004, Japan; Department of Neurology and Stroke Medicine, Graduate School of Medicine, Yokohama City University, Yokohama 236-0004, Japan; Department of Neurology, Yokohama Brain and Spine Center, Yokohama 235-0012, Japan; Department of Neurology and Stroke Medicine, Graduate School of Medicine, Yokohama City University, Yokohama 236-0004, Japan; Department of Neurology and Stroke Medicine, Graduate School of Medicine, Yokohama City University, Yokohama 236-0004, Japan; Department of Neurology and Stroke Medicine, Graduate School of Medicine, Yokohama City University, Yokohama 236-0004, Japan; Department of Neurology and Stroke Medicine, Graduate School of Medicine, Yokohama City University, Yokohama 236-0004, Japan; Department of Neurology, Yokohama City University Medical Center Hospital, Yokohama 232-0024, Japan; Department of Neurology and Stroke Medicine, Graduate School of Medicine, Yokohama City University, Yokohama 236-0004, Japan; Department of Neurology, Yokohama City University Medical Center Hospital, Yokohama 232-0024, Japan; Department of Neurology and Stroke Medicine, Graduate School of Medicine, Yokohama City University, Yokohama 236-0004, Japan; Department of Neurology, Yokohama City University Medical Center Hospital, Yokohama 232-0024, Japan; Department of Neurology and Stroke Medicine, Graduate School of Medicine, Yokohama City University, Yokohama 236-0004, Japan; Department of Neurology, Yokohama Brain and Spine Center, Yokohama 235-0012, Japan; Department of Neurology and Stroke Medicine, Graduate School of Medicine, Yokohama City University, Yokohama 236-0004, Japan

**Keywords:** cerebellum, cerebellar cognitive affective syndrome, Poggendorff illusion, internal model

## Abstract

This study aimed to test our hypothesis that the cerebellum plays an important role in the generation of the optical-geometric illusion known as the Poggendorff illusion, the mechanism of which has been explained by accumulated experience with natural scene geometry. A total of 79 participants, comprising 28 patients with isolated cerebellar stroke, 27 patients with isolated cerebral stroke and 24 healthy controls, performed Poggendorff illusion tasks and 2 different control tasks. We also investigated core brain regions underpinning changes in the experience of the illusion effect using multivariate lesion-symptom mapping. Our results indicate that patients with isolated cerebellar stroke were significantly less likely to experience the Poggendorff illusion effect than patients with isolated cerebral stroke or healthy controls (74.6, 90.5 and 89.8%, respectively; *F*(2,76) = 6.675, *P* = 0.002). However, there were no inter-group differences in the control tasks. Lesion-symptom mapping analysis revealed that the brain lesions associated with the reduced frequency of the Poggendorff illusion effect were mainly centred on the right posteromedial cerebellar region, including the right lobules VI, VII, VIII, IX and Crus II. Our findings demonstrated, for the first time, that patients with cerebellar damage were significantly less likely to experience the Poggendorff illusion effect and that right posteromedial cerebellar lesions played an important role in this effect. These results provide new insight into alterations of a geometric illusion effect in patients with cerebellar disorders and pave the way for future clinical use of the illusion task to detect cerebellar abnormalities.

## Teaser

Higashiyama *et al*. identified, for the first time, that patients with an isolated cerebellar stroke had a significantly reduced likelihood of experiencing the Poggendorff illusion. As an anatomical basis for this finding, Higashiyama *et al*. demonstrated the significance of right posteromedial cerebellar lesions.

## Graphical Abstract

**Graphical abstract:**
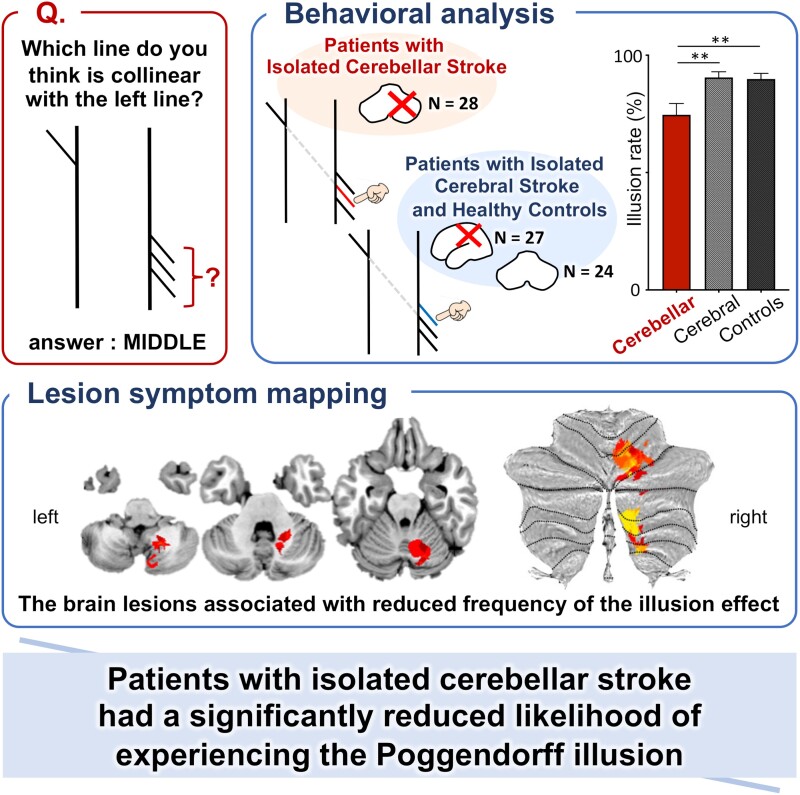

## Introduction

The cerebellum, known as one of the centres for motor control, is increasingly understood to play a broad role in cognitive functions. Anatomical studies in monkeys and functional imaging studies in humans have shown that the cerebellum has both afferent and efferent connections with regions of the cerebral cortex other than the primary motor cortex, such as the parietal association cortex and the prefrontal cortex.^1,2^ Additionally, a growing number of human lesion studies suggest cerebellar involvement in various types of cognitive dysfunction, collectively referred to as ‘cerebellar cognitive affective syndrome (CCAS)’^3^ or Schmahmann’s syndrome.^4^ Recently, CCAS has been identified in many focal and diffuse diseases of the cerebellum, both acquired and inherited.^5,6^ However, qualitative differences between cognitive impairment caused by cerebellar versus cerebral or other subcortical lesions remain unknown.

As for the pathophysiology of cognitive function associated with the cerebellum, the ‘internal model hypothesis’ was proposed regarding mental activities.^7^ Through a motor learning process, the cerebellum forms an internal model that helps the brain to perform the movement precisely without the need to refer to feedback from the moving body part.^7^ It is broadly assumed that cerebellar dysfunction leads to a failure to generate proper motor commands or to estimate the precise consequences of such commands through internal models. Regarding cognitive functions of the cerebellum, it is probable that internal models work in the same way as those involving movement control, because the cerebellum has a homogenous microstructure and rich connections with the frontal and parietal association cortices. Considering that CCAS might be caused by a disruption of the internal model, tasks related to empirically learned perception could be used to evaluate cerebellum-specific cognitive function. From this point of view, we focused on an optical-geometric illusion named the Poggendorff illusion.

**Figure 1:**
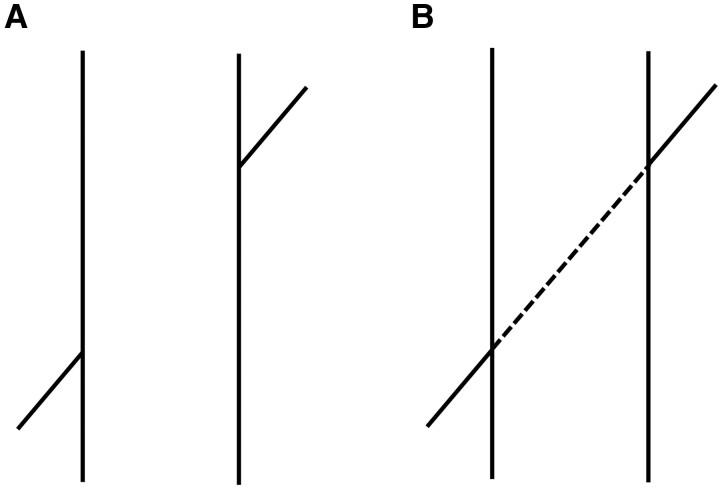
**Poggendorff illusion.** (**A)** Collinear oblique lines, separated by two vertical lines, are erroneously perceived as misaligned. (**B**) Addition of a dashed line between the two vertical lines in **A**.

In the 19th century, Johann Poggendorff pointed out that when two collinear, obliquely oriented lines are interrupted by two parallel lines, the oblique lines no longer appear collinear and instead one appears to be shifted vertically (Fig. 1). Although this figure is one of the best-known and most extensively investigated optical-geometric illusions, there is no consensus about its mechanism. However, Howe *et al*.^8,9^ provided the ingenious hypothesis that this illusion is generated by accumulated experience with natural scene geometry. They analysed a large database of environmental images and showed that the Poggendorff figure resembles objects in these images. Specifically, they found evidence suggesting that past ‘experience’ in the natural environment creates the illusion. If this is true, the Poggendorff illusion must be associated with the cerebellum, because the internal model plays an important role in the automatization of learned skills.

Based on these findings, we hypothesized that the cerebellum plays a crucial role in the generation of the Poggendorff illusion and that cerebellar impairment should abolish or reduce the illusion effect. Accordingly, we conducted a clinical and neuroimaging study to investigate the relationship between isolated cerebral or cerebellar lesions and the Poggendorff illusion effect.

## Materials and methods

### Participants and behavioural assessment

Seventy-nine participants were recruited for this study between May 2014 and March 2017: 28 patients with a first stroke confined to the cerebellum (cerebellar stroke: CS group), including 24 ischaemic infarctions and 4 haemorrhages; 27 patients with a first stroke confined to the cerebrum (non-cerebellar stroke: NCS group), including 17 ischaemic infarctions and 10 haemorrhages; and 24 age-, sex- and education-matched healthy controls (HCs). All patients were recruited from Yokohama City University Hospital and Yokohama City Stroke and Spine Center, and all healthy individuals were recruited from the local community. Inclusion criteria for all participants were (i) right-handedness according to the Edinburgh Handedness Inventory; (ii) normal or corrected-to-normal vision without double vision, restricted eye movement, hemianopia or unilateral neglect; and (iii) no severe cognitive/psychiatric impairment. The inclusion criteria for patients with stroke were: (i) diagnosis of first-ever stroke (ischaemic or haemorrhagic) confirmed by CT or MRI, (ii) lesions completely localized to either the cerebrum or the cerebellum and (iii) lack of pre-existing structural degenerative changes (e.g. severe brain atrophy and extensive white matter changes). Furthermore, we excluded participants who failed to respond correctly to the control tasks described below (cut-off score ≤33%) because they seemed unlikely to be able to perform our illusion task (six patients were excluded: two patients from the CS group and four patients from the NCS group). The Mini-Mental State Examination (MMSE) was performed in all participants, and all participants in the CS group underwent neurological examination and evaluation of cerebellar motor function by the Scale for the Assessment and Rating of Ataxia (SARA)^10^ (Table 1).

**Table 1:** Clinical and demographic data of patients and controls

Characteristic		Cerebellar stroke (*n* = 28)	Non-cerebellar stroke (*n* = 27)	Healthy control (*n* = 24)	*P*-value	Effect size	*Post hoc*
Age (years)		69.6 (13.4)	66.3 (11.2)	67.5 (13.2)	0.618	*η* ^2^ = 0.013	
Sex (M/F)		19/9	15/12	13/11	0.530	*V* = 0.127^a^	
Durations (days)							
Acute to subacute (<3 weeks)	*N*	18	24				
	Days	7.3 (4.5)	6.5 (4.6)		0.406	*r* = 0.128	
Chronic (≥3 weeks)	*N*	10	3				
	days	901 (1436)	500 (759)		0.937	*r* = 0.047	
MMSE		26.6 (3.6)	26.3 (3.9)	29.3 (1.8)	0.003	*η* ^2^ = 0.139	HC > CS, NCS
SARA		5.89 (7.4)					
Lesion volume (ml)		21.7 (23.2)	25.2 (35.2)		0.876	*r* = 0.021	

Data are given as mean (standard deviation). CS, cerebellar stroke; HC, healthy controls; MMSE, Mini-Mental State Examination; NCS, non-cerebellar stroke; SARA, scale for the assessment and rating of ataxia. ^a^Cramer’s *V*.

The study was approved by the local ethics committee of Yokohama City University and Yokohama City Stroke and Spine Center. All participants gave written informed consent prior to participation, according to the Declaration of Helsinki.

### Experimental design

**Figure 2:**
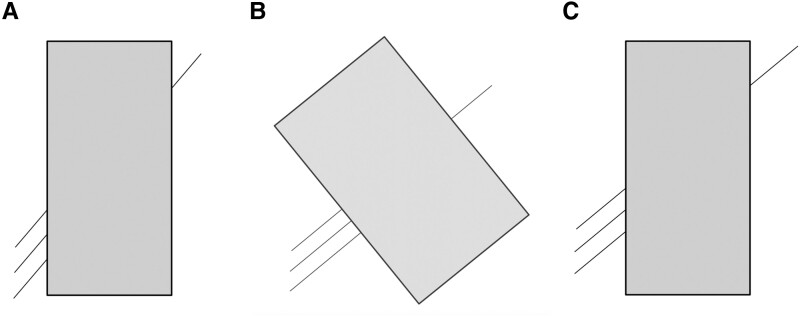
**Visual illusion tasks.** (**A**) An example of a Poggendorff illusion figure. There are three target lines on the left side of a rectangle and one seed line on the right side. If the seed line is extended, it reaches the middle target line; i.e. ‘the middle line’ is the correct answer. However, most healthy participants choose the upper line, a response that demonstrates the Poggendorff illusion effect. (**B**) An example of an oblique control figure. The correct answer is ‘the upper line.’ (**C**) An example of an upper control line. The correct answer is ‘the upper line’.

The Poggendorff illusion task comprised nine figures with different line lengths and directions, in reference to previous studies.^8,11^ Each figure consisted of one line on one side (seed line), three lines on the other side (target lines) tilted at the same angle as the seed line, and a grey rectangle interrupting the seed line and the three target lines (Fig. 2A). One of the three target lines was collinear with the seed line, and participants were instructed to identify the collinear target line (i.e. upper, middle or lower). If a participant chose a line that was shifted upward with respect to the correct line, this indicated the Poggendorff illusion effect.

In addition, we adopted two control figures (oblique and upper) to assess basic visual function and general attention. In the oblique control figure, the grey rectangle was tilted at a 90° angle relative to the seed line (Fig. 2B). It is well documented that the illusion effect disappears when the transverse line forms a 90° angle with the rectangle,^12,13^ and therefore, the effect should be absent in the oblique control figure. With this figure, it was assumed that the visual completion phenomenon^14^ would make it easy for healthy participants to correctly identify the geometrically collinear line. Regarding the upper control figure, the participant should choose the upper line regardless of whether they experienced the illusion effect with non-control figures (Fig. 2C). As described above, the oblique control figures were implemented to exclude participants whose basic visual function or general attention was insufficient to complete the illusion task.

The visual stimuli consisted of nine Poggendorff figures and six control figures (three oblique and three upper), each of which was randomly presented on a piece of paper positioned 60 cm in front of a comfortably seated participant. The participant was instructed to identify and report verbally the target line that was collinear with the seed line and to select that target line as their answer. Participants were not allowed to use their fingers or to rotate the paper. These tests were performed by a trained neurologist (Y.H.) and a trained occupational therapist (M.K.) to ensure standardized data collection.

Regarding the Poggendorff illusion task, we calculated the percentage of trials in each group that resulted in the Poggendorff illusion effect (Poggendorff illusion rate). We also calculated the rate of correct responses for each control figure.

### Statistical analysis

Normality and homogeneity were checked for all clinical and demographic variables using the Shapiro–Wilk test and Levene’s tests, respectively. We found that some data were normally distributed, but some were not. Considering the robustness of one-way ANOVA against violations of normality and its better control of Type I error compared with non-parametric equivalents,^15^ we employed ANOVA followed by Tukey’s honest significance test to compare pair or multiple groups means for age, MMSE and the results of all visual tasks. Lesion volumes and disease durations were assessed using the Mann–Whitney U-test. Sex was assessed using the *χ*^2^ test. Associations between demographic, clinical and cognitive variables and the Poggendorff illusion rate were analysed using Spearman’s rank correlations. Effect sizes were calculated by *r* scores or *η*^2^, as appropriate. All statistical analyses were performed with IBM SPSS Statistics version 22.0 (the significance level was *α* = 0.05).

### Neuroimaging analysis

In the imaging analysis, one patient with cerebral haemorrhage in the NCS group was excluded due to a lack of MRI data (only CT data were available). Therefore, 54 patients, comprising 28 and 26 patients with CS and NCS, respectively, underwent 3.0T MRI (Ingenia, Philips Healthcare, Best, The Netherlands) or 1.5T MRI (Signa Explorer; GE Medical Systems, Milwaukee, WI, USA) within 2 weeks before or after neuropsychological evaluation, and axial T1, T2 or fluid-attenuated inversion recovery and diffusion weighted imaging (*b* = 1000 s/mm^2^) images were obtained for lesion analysis and spatial normalization (slice thickness = 6 mm).

Lesions on each patient’s original T2 or fluid-attenuated inversion recovery images were demarcated manually using MRIcron (http://www.mccauslandcenter.sc.edu/mricro/mricron/), with reference to the diffusion weighted images of patients in the acute to subacute phase (see Supplementary Table 1 for more details regarding the reference MRI modalities); this was done by a trained neurologist (Y.H.) and an occupational therapist (M.K.) by masking any reference information other than the MRI data. Using the normalization algorithm of SPM12 (http://www.fil.ion.ucl.ac.uk/spm/) and the Clinical Toolbox for SPM12, original lesion masks were normalized to a standard T1 template with a resampled voxel size of 1 × 1 × 1 mm^3^.^16^ The precision of spatial normalization was ensured by visual inspection with reference to standard templates (we found no error in this process for any subject). Then, the normalized lesion map was analysed using support vector regression–based multivariate lesion-symptom mapping (SVR-LSM) to identify the statistical contribution of lesion location to the Poggendorff illusion rate in the CS and NCS groups. SVR-LSM has been used and validated as a multivariate method to model lesion-symptom associations in multiple studies.^17–22^ Contrary to standard mass-univariate voxel-based lesion-symptom mapping (VLSM), which assumes statistical independence across voxels, SVR-LSM uses a high-dimensional feature space to simultaneously evaluate the entire brain–behaviour association and provides better sensibility and specificity.^22^ In this study, we used DeMarco and Turkeltaub’s toolbox^17^ to apply functionalities of the Statistics and Machine Learning Toolbox within MATLAB (MATLAB 2022a; The MathWorks, Inc., Natick, MA, USA). The following settings were used in accordance with previous studies^17,21^: MATLAB Support Vector Machine implementation with default parameters gamma = 5 and cost = 30, minimum lesion overlap = 3, 10 000 permutations, voxel-wise *P* < 0.005 and cluster-wise *P* < 0.05, and age, sex and time post-stroke as covariates regressed out of behavioural scores. To control for lesion volume effects, we adopted the direct total lesion volume control option in the SVR-LSM toolbox.^17,22^ However, since traditional mass-univariate methods are still widely used, we replicated our analysis using VLSM to confirm the robustness of the results across methodological approaches (detailed methods and results are included in the Supplementary materials). To determine damaged brain regions, affected voxels were overlaid on the Automated Anatomic Labeling atlas^23^ or the Johns Hopkins University atlas.^24^ The lobules and cerebellar nuclei involved in each cluster were identified using the Spatially Unbiased Infratentorial (SUIT) atlas.^25^ The resultant maps were also converted into a surface representation using the SUIT flat map for visualization purposes.^26^

## Results

### Participants and behavioural assessment

Table 1 shows the participant characteristics. There were no significant differences between groups in terms of age, sex or lesion volume (Table 1). Regarding disease duration, the CS group included 18 patients in the acute-to-subacute phase (within 3 weeks after onset) and 10 patients in the chronic phase (>3 weeks after onset). The NCS group included 24 and 3 cases in the acute-to-subacute and chronic phases, respectively. While the CS group included a relatively higher number of chronic-phase patients (*P* = 0.06 using the *χ*^2^ test), there were no significant differences between groups in terms of disease duration within each phase. The only difference between groups was in the MMSE score (*F*(2,76) = 6.151, *P* = 0.003). In *post hoc* analyses, the CS and NCS groups revealed significant impairment compared with the HC group (*P* = 0.013 and 0.005, respectively). A comparison analysis of the acute and chronic patient groups showed no significant differences in age, gender, MMSE or lesion volume, although SARA scores were significantly higher in the chronic group. Detailed data on patients in the acute-to-subacute phase or the chronic phase are presented in Supplementary Table 2.

### Poggendorff illusion task and control tasks

**Figure 3:**
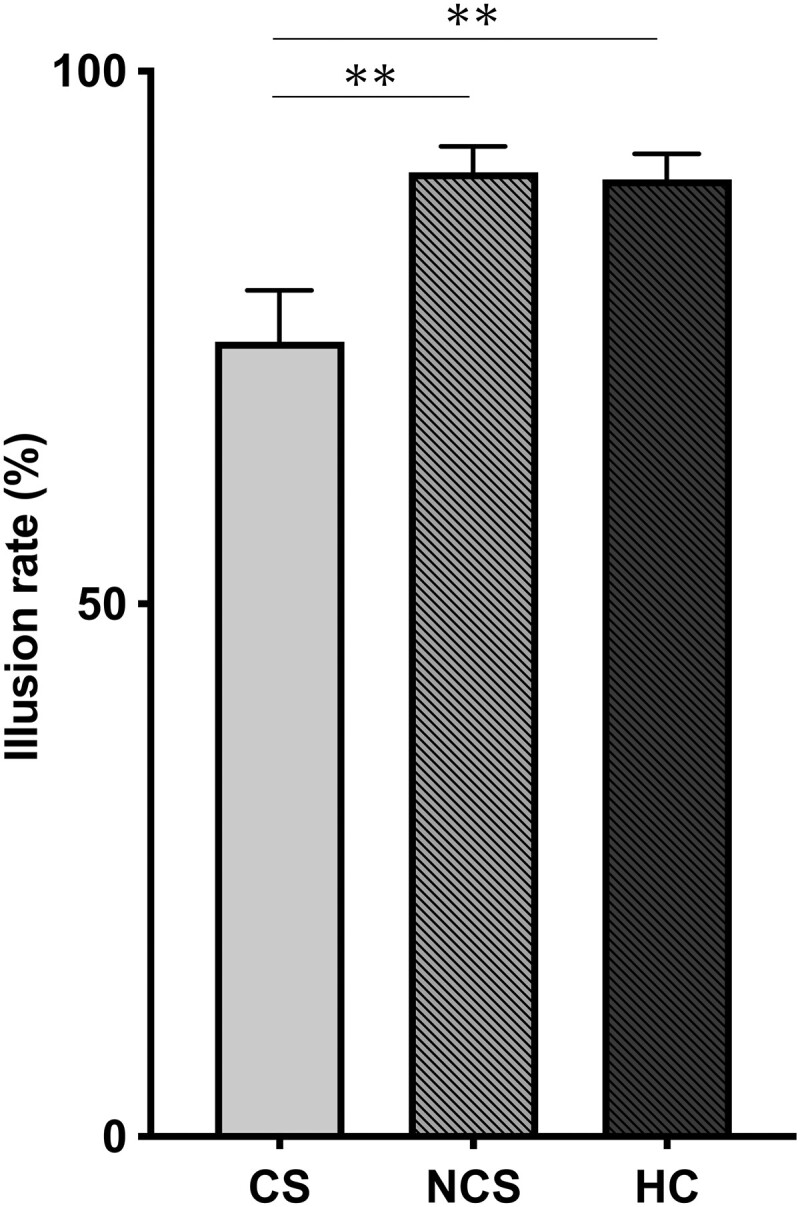
**Poggendorff illusion rates.** Average Poggendorff illusion rates for all participants are shown with error bars denoting ±1 standard error of the mean. The CS group shows a lower Poggendorff illusion rate than the NCS and HC groups (one-way ANOVA). CS, cerebellar stroke; NCS, non-cerebellar stroke; HC, healthy controls. **P < 0.01.

Table 2 illustrates the results of all tasks. The Poggendorff illusion rates of the CS, NCS and HC groups were 74.6 ± 25.6, 90.5 ± 13.0 and 89.8 ± 11.8, respectively (mean ± standard deviation), showing a significant difference among the groups (*F*(2,76) = 6.675, *P* = 0.002). According to *post hoc* analysis, the CS group had a lower Poggendorff illusion rate than the NCS group (*P* = 0.005) and HC group (*P* = 0.010) (Fig. 3). This means that the CS group was significantly more likely than the other groups to correctly choose the target line that was collinear with the seed lines. Regarding the control tasks, the results of the oblique control task (*F*(2,76) = 0.511, *P* = 0.602) and the upper control task (*F*(2,76) = 0.962, *P* = 0.387) did not differ between groups. Even when we excluded chronic-phase patients from the analysis, the CS group still showed a significantly lower Poggendorff illusion rate than the NCS group (*P* = 0.049) and HC group (*P* = 0.049) (Supplementary Fig. 1 and Table 3). In addition, there was no significant difference in the Poggendorff illusion rate between the acute-to-subacute and chronic phases in the CS group (*t*(26) = 0.530, *P* = 0.601). Correlation analysis and group comparison analysis between the illusion effect and each clinical characteristic (age, sex, aetiology, MMSE, SARA, lesion volume and disease duration) showed no significant results in the CS group alone or in both stroke groups combined (CS and NCS; Supplementary Tables 4 and 5).

**Table 2:** Visual illusion task scores of patients and controls

Task	Cerebellar stroke (*n* = 28)	Non-cerebellar stroke (*n* = 27)	Healthy control (*n* = 24)	*F*-value (df = 78)	*P*-value	Effect size (*η*^2^)	*Post hoc*
Poggendorff task (illusion rate %)	74.6 (25.6)	90.5 (13.0)	89.8 (11.8)	6.675	0.002	0.149	HC, NCS > CS
Oblique control task (correct rate %)	91.7 (14.7)	95.1 (12.1)	94.4 (12.7)	0.511	0.511	0.013	–
Upper control task (correct rate %)	100 (0.0)	98.8 (6.4)	100 (0.0)	0.962	0.387	0.025	–

Date are given as mean (standard deviation). CS, Cerebellar stroke; df, degrees of freedom; HC, healthy controls; NCS, non-cerebellar stroke.

### Neuroimaging analysis

**Figure 4:**
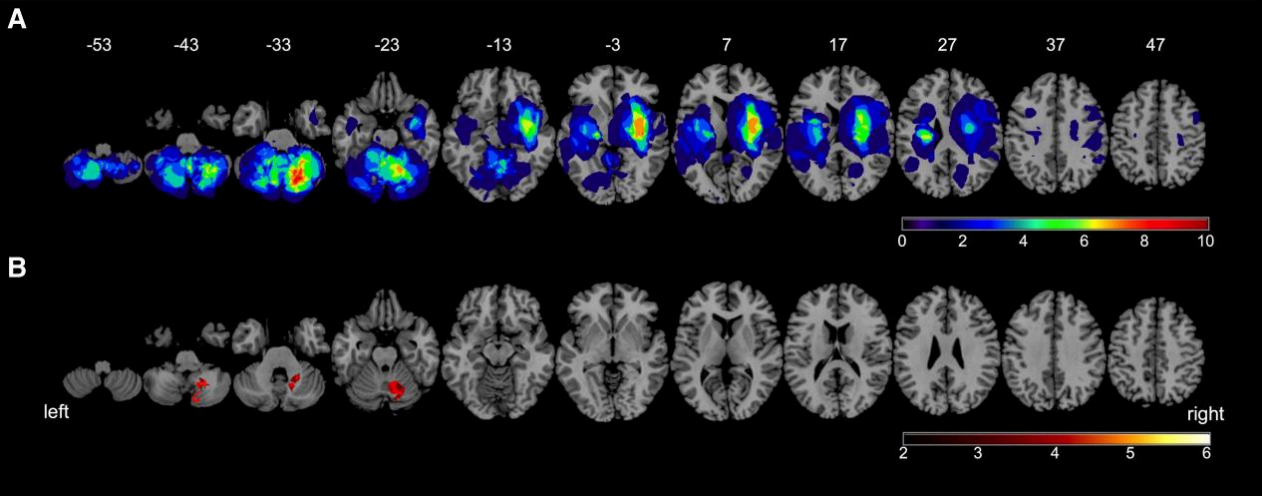
**Lesion overlap maps and SVR-LSM results.** (**A**) Lesion overlap maps for all patients. The colour scale indicates the relative number of patients with overlapping lesions. (**B**) Results of SVR-LSM. Lesioned areas associated with significantly reduced Poggendorff illusion rates are shown. The colour scale indicates the *Z* score. The results are thresholded at voxel-wise *P* < 0.005 and corrected for cluster size at *P* < 0.05 based on 10 000 permutations. Data were corrected for lesion volume, sex, age and disease duration, and the maximum voxel value was *Z* = 3.719 (Montreal Neurological Institute coordinates: 22, −59, −43).

**Figure 5:**
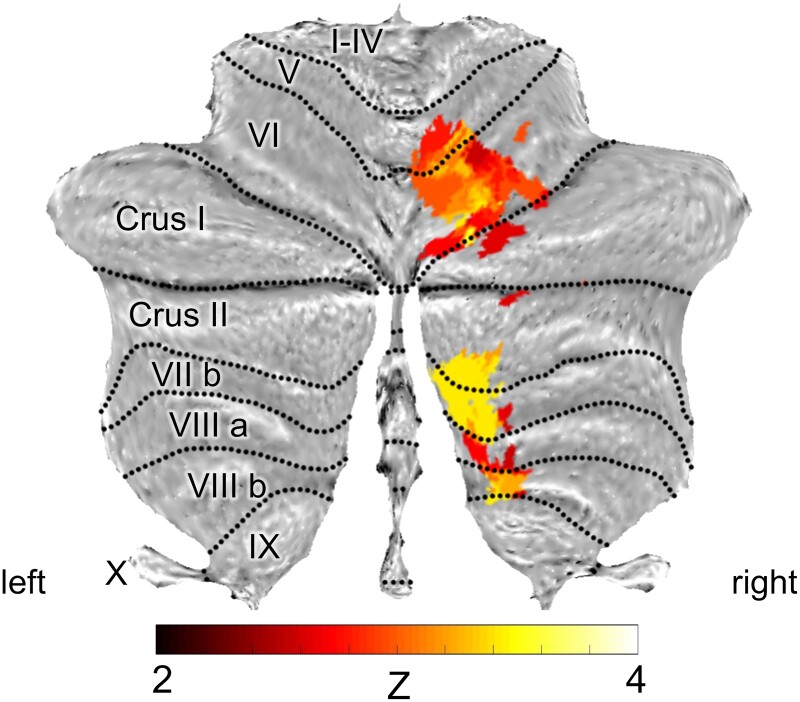
**Results of SVR-LSM on the cerebellar flat map.** Lesioned areas associated with significantly reduced Poggendorff illusion rates are visualized on a cerebellar flat map using the SUIT toolbox for SPM. The colour scale indicates the *Z* score. The results are thresholded at voxel-wise *P* < 0.005 and corrected for cluster size at *P* < 0.05 based on 10 000 permutations. Data were corrected for lesion volume, sex, age and disease duration, and the maximum voxel value was *Z* = 3.719 (Montreal Neurological Institute coordinates: 22, −59, −43).

Figure 4A demonstrates the lesion overlap maps for all patients with stroke, and Figures 4B and 5 show the results of SVR-LSM analyses. The brain lesions associated with a reduced Poggendorff illusion rate were centred on the right posteromedial cerebellar region. In reference to the cerebellar flat map, significant clusters of lesions are located in right lobules VI, VII, VIII, IX and Crus II. However, there were no significant clusters in the cerebellar nuclei or cerebral regions. The clusters of cerebellar regions demonstrating a reduced Poggendorff illusion rate were nearly identical to those derived from the results of the traditional mass-univariate method, i.e. VLSM (Supplementary Figs 3 and 4). The main difference was that VLSM analysis identified larger and more distributed clusters in right lobule IX (Supplementary Fig. 4).

Even when we excluded chronic-phase patients from the analysis, lesions associated with reduced Poggendorff illusion rates were also centred in the right posteromedial cerebellar region, but with slightly more widespread involvement that still included the same lobules (Supplementary Fig. 2).

## Discussion

### Reduced Poggendorff illusion rate in the cerebellar stroke group

To test our hypothesis that the cerebellum plays a crucial role in generating the Poggendorff illusion effect, we evaluated this effect in patients with cerebral stroke and CS. Consistent with our hypothesis, patients in the CS group showed a significantly lower illusion rate than those in the other groups.

It is possible that the CS group had impairments in general attention or basic visual function, including saccade dysmetria and nystagmus, which affected their choices in the illusion task. In practical terms, however, such concerns can be dismissed because the CS group showed similar results to the other groups in all control tasks (Table 2). These tasks do not elicit the illusion effect and are regarded as simple visuospatial tasks. Accordingly, our results imply that the CS group had similar visuospatial and general attentional functioning as the NCS and HC groups.

A variety of theories have been proposed to account for the Poggendorff illusion. One is that the viewer experiences a misperception of the angles in the stimulus.^27^ The ‘depth-processing’ theory states that the oblique lines in the Poggendorff stimulus are interpreted as lines extending in depth and are therefore perceived to be non-collinear.^28,29^ However, these theories are limited because they cannot explain all aspects of the illusion and its variants.^8^ On the other hand, Howe *et al*. hypothesized that the Poggendorff illusion and its variants are rooted in the frequency of real-world images, resembling the Poggendorff figure. Using a database containing a large series of images of natural scenes with 3D structure information, the authors demonstrated that the Poggendorff illusion and all of its variants were fully explained by the probability distribution of the possible locations of line segments across intervals in images of the natural environment. They concluded that the Poggendorff illusion results from the empirical examination of the natural environment rather than from the principles of projective geometry *per se.*^8,9^ Our results supported this view and are consistent with the fact that the cerebellum has been empirically regarded as necessary for learning new skills.

The neuronal circuitry of the cerebellum is thought to encode internal models that must be continuously modified and updated through learning processes that compare their output with the output of the controlled object.^7^ Using these internal models, the cerebellum facilitates precise and skilful movements without feedback from the moving body part. Thus, empirically learned feed-forward motor control can be regarded as a key cerebellar function. Unlike the cerebral cortex, the cerebellum has a homogeneous cytoarchitecture and modular circuitry consisting of two major fibres (i.e. climbing fibres and mossy fibres).^7^ These anatomical and physiological uniformities of cortico-nuclear microcomplexes provide the basis for consistent cerebellar computation.^30^ However, recent anatomical and functional neuroimaging studies also revealed that the cerebellum is linked in a reciprocal manner not only with the motor-sensory cortex but also with the limbic system and its associative regions in the supratentorial cortex.^2^ Considering both the anatomical uniformities and the rich connections between the cerebellum and various cerebral regions, it is supposed that movement, cognition and emotion share a single neurological process that utilizes the internal model involved in cerebellar modulation. Therefore, cerebellar damage might cause specific cognitive impairments, which like cerebellar motor symptoms, are characterized by dysmetria and decomposition.

In this respect, we presume that the reduced Poggendorff illusion rate in our cerebellar patients might be attributed to disruption of the internal model related to visuospatial cognition, in accordance with the hypothesis that the Poggendorff illusion is generated by accumulated experience of natural scene geometry.^8^ Supporting evidence for the role of the cerebellum in visuospatial perception also derives from experimental findings showing the importance of cerebellar circuits for acquiring the procedural components required for spatial learning.^31,32^ Further, clinical studies demonstrated that cerebellar lesions affect visuospatial ability as assessed by the mental rotate task.^33^ However, to our best knowledge, this is the first report showing changes in a geometric illusion effect in relation to cerebellar impairments. Further studies should confirm the role of the cerebellum in empirically learned geometric skills and elucidate the mechanisms underpinning geometric illusions. Such studies should ideally target other cerebellar disorders using detailed tasks to measure illusion magnitude or focus on other geometric illusions, such as the Müller–Lyer illusion.

### Relationships between the illusion effect and other clinical factors

Regarding the duration since stroke onset, the CS group showed a lower illusion rate than the NCS and HC groups, irrespective of disease phase. It was reported that CCAS was less evident in patients with stroke during the recovery phase (3–4 months),^34^ which is inconsistent with our results. However, another study supported our results, revealing that patients with chronic CSs still showed significant CCAS.^21^ In any case, since our NCS cohort contained a very small number of patients in the chronic phase, future large-scale studies involving patients in various disease phases may clarify the relationship between disease course and the reduced illusion rate.

The reduced illusion rate had no correlation with motor symptoms as evaluated by SARA. This finding is concordant with previous findings that CCAS can exist in the absence of motor deficits.^35,36^ However, a limitation of our study is the lack of detailed cognitive evaluations on CCAS. The MMSE is insufficient to evaluate detailed cognitive functions; this includes executive functions, which comprise one of the cognitive domains most affected by cerebellar injury. To clarify the relationship between the reduced illusion rate and CCAS, further studies should include intensive neuropsychological batteries and various illusion tasks.

### Relation between the posteromedial cerebellar region and the reduced Poggendorff illusion rate

In our SVR-LSM analysis, reduced Poggendorff illusion rates were regionally associated primarily with the right posteromedial cerebellar areas, including the right lobules VI, VIIb, VIII, IX and Crus II (Figs 4B and 5), indicating that these regions may be responsible for the illusion effect.

Previous anatomical studies in animals have shown cerebellar subspecialization and corresponding neural connections with the cerebral cortex: the anterior cerebellum connects with the primary motor cortex, and posterior cerebellar areas connect with the pre-frontal and parietal cortex (non-motor cortex).^1,37^ Functional neuroimaging studies in healthy human volunteers have also shown this dichotomy. Two meta-analyses and one study using a large task-based functional MRI data set showed that cerebellar activation patterns were task dependent; sensorimotor tasks activated the motor cerebellar region, while cognitive and emotional tasks activated non-motor cerebellar regions.^38-40^ Additionally, studies have consistently reported that spatial processing tasks activate posteromedial cerebellar lobules VI and VII.^38,41^ Furthermore, a resting-state functional MRI study focusing on intrinsic cerebro-cerebellar networks showed that the posteromedial clusters identified in our study were mainly involved in the ventral/dorsal attention network.^42^ These results strongly suggest that the region in which our clusters are located plays a crucial role in the empirical learning process of visuospatial attention that is related to the development of the Poggendorff illusion.

With respect to hemispheric laterality, we predicted that relevant clusters would be centred in the left cerebellum because previous task-based functional neuroimaging studies demonstrated greater left-cerebellar, hemisphere-dominant activation in visuospatial processing tasks.^38,39^ Contrary to our expectations, all clusters were localized in the right cerebellar hemisphere. It was possible that selection bias occurred, involving the inclusion of a disproportionately high number of right cerebellar hemispheric cases. However, the lateralization of visuospatial processing tasks in previous functional neuroimaging studies was reported to be less consistent than that of language tasks. For instance, a spatial navigation task engaged a cluster in the right Crus I,^43^ and the bilateral cerebellar hemispheres were activated during the encoding of shape and colour combinations in a paired-associate learning task.^44^ Lee *et al*.^45^ also reported bilateral cerebellar activation in a modified version of Benton’s Judgment of Line Orientation, which like our illusion task, assesses geometric visuospatial abilities. Previous lesion studies in humans also provided support for our results. Stoodley *et al*.^36^ reported that impairment in Rey figure copy accuracy and performance on Benton’s Judgment of Line Orientation were associated with damage to a large cluster extending from the right VIIb into VIIIb and regions in the right VIIIb/IX, respectively, both adjacent to the cerebellar regions identified in our SVR-LSM results. These findings suggest that the right posteromedial cerebellum plays an important role in cognitive processes related to the perception of geometric figures.

## Conclusion

This study identified for the first time a significantly reduced Poggendorff illusion rate in cerebellar patients and demonstrated the importance of right posteromedial cerebellar lesions. Our results offer novel insights regarding cerebellar contributions to an optical-geometric illusion effect.

## Supplementary Material

fcad053_Supplementary_Data

## Data Availability

The data that support the findings of this study are available from the corresponding author upon reasonable request.

## Abbreviations

CCAS: cerebellar cognitive affective syndrome
CS: cerebellar stroke
HC: healthy control
MMSE: Mini-Mental State Examination
NCS: non-cerebellar stroke
SARA: Scale for the Assessment and Rating of Ataxia
SUIT: Spatially Unbiased Infratentorial
SVR-LSM: support vector regression–based multivariate lesion-symptom mapping
VLSM: voxel-based lesion-symptom mapping
