# Supplementary material for: Reduced likelihood of the Poggendorff illusion in cerebellar strokes: a clinical and neuroimaging study
Source: Brain Commun. 2023 Mar 6;5(2):fcad053. doi: 10.1093/braincomms/fcad053 (PMC10018644; doi:10.1093/braincomms/fcad053)
Supplement: fcad053_Supplementary_Data [file fcad053_supplementary_data.zip › Supplementary_material_figures_and_legends.docx]

**Supplementary Table 1 - Clinical data and lesion locations in the patients with stroke**

| ID | etiology | days after the stroke | illusion rate (%) | brain lesions | Refference MRI modalities |
| --- | --- | --- | --- | --- | --- |
| #01 | hemorrhage | 2611 | 66.7 | 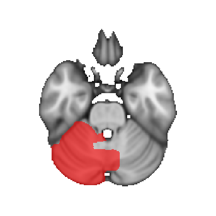  left  right | FLAIR |
| #02 | Hemorrhage | 255 | 44.4 | 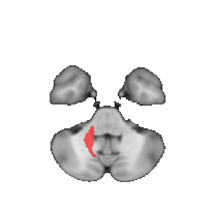 | T2WI & DWI |
| #03 | Hemorrhage | 8 | 33.3 | 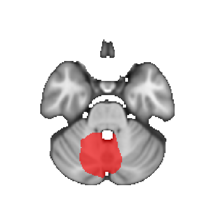 | FLAIR & DWI |
| #04 | hemorrhage | 8 | 100.0 | 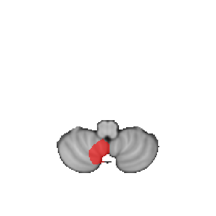 | FLAIR & DWI |
| #05 | infarction | 29 | 44.4 | 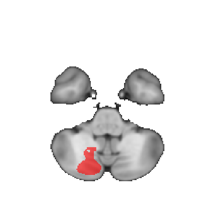 | FLAIR & DWI |
| #06 | infarction | 1015 | 77.8 | 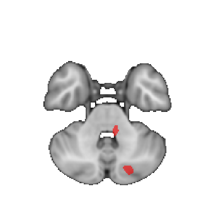 | FLAIR |
| #07 | Infarction | 19 | 100.0 | 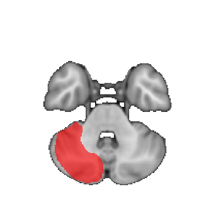 | FLAIR & DWI |
| #08 | Infarction | 4304 | 55.6 | 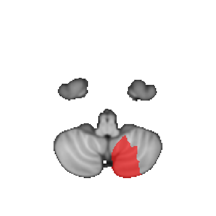 | FLAIR |
| #09 | infarction | 6 | 66.7 | 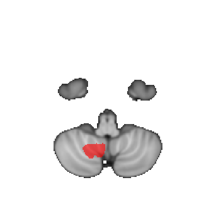 | FLAIR & DWI |
| #10 | infarction | 7 | 88.9 | 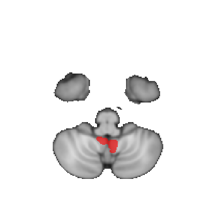 | FLAIR & DWI |
| #11 | Infarction | 369 | 77.8 | 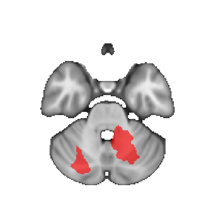 | T2WI |
| #12 | infarction | 23 | 44.4 | 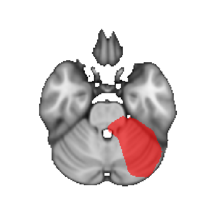 | FLAIR & DWI |
| #13 | infarction | 6 | 100.0 | 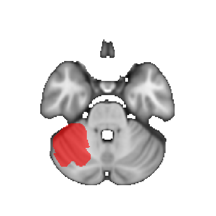 | T2WI & DWI |
| #14 | infarction | 7 | 55.6 | 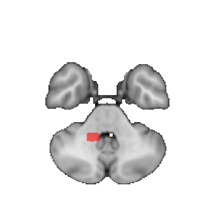 | FLAIR & DWI |
| #15 | infarction | 3 | 88.9 | 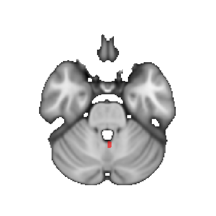 | FLAIR & DWI |
| #16 | infarction | 15 | 44.4 | 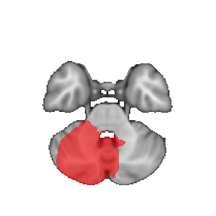 | T2WI & DWI |
| #17 | infarction | 349 | 100.0 | 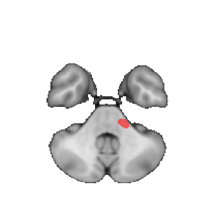 | FLAIR |
| #18 | infarction | 5 | 22.2 | 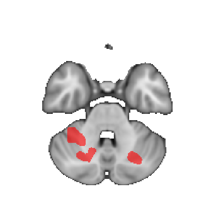 | FLAIR & DWI |
| #19 | infarction | 3 | 44.4 | 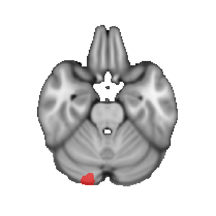 | T2WI & DWI |
| #20 | infarction | 6 | 100.0 | 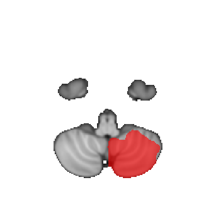 | T2WI & DWI |
| #21 | infarction | 33 | 100.0 | 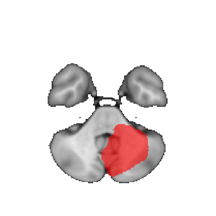 | FLAIR & DWI |
| #22 | infarction | 2 | 100.0 | 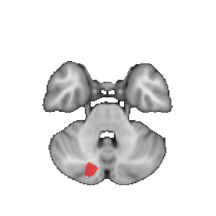 | FLAIR & DWI |
| #23 | infarction | 7 | 100.0 | 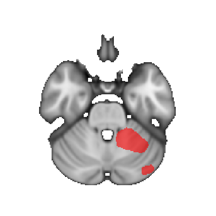 | T2WI & DWI |
| #24 | infarction | 13 | 55.6 | 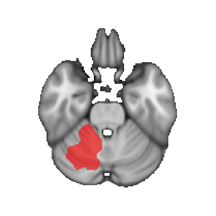 | T2WI & DWI |
| #25 | infarction | 9 | 77.8 | 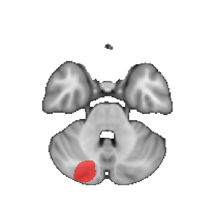 | T2WI & DWI |
| #26 | infarction | 27 | 100.0 | 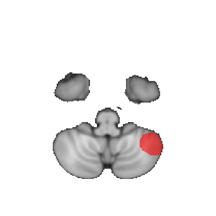 | FLAIR & DWI |
| #27 | infarction | 2 | 100.0 | 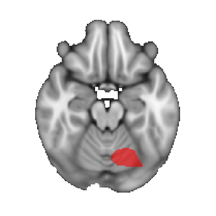 | T2WI & DWI |
| #28 | infarction | 6 | 100.0 | 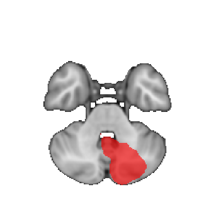 | FLAIR & DWI |
| #29 | hemorrhage | 8 | 100.0 | 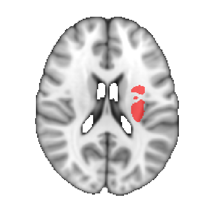 | T2WI & T1WI & DWI |
| #30 | hemorrhage | 1376 | 100.0 | 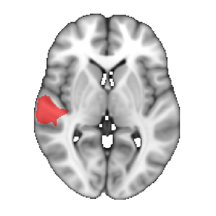 | FLAIR & T1WI |
| #31 | hemorrhage | 3 | 77.8 | 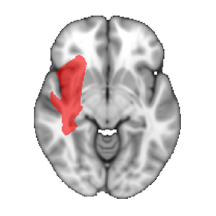 | FLAIR & T1WI & DWI |
| #32 | hemorrhage | 20 | 77.8 | 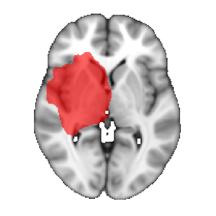 | FLAIR & T1WI & DWI |
| #33 | hemorrhage | 9 | 77.8 | 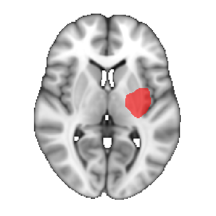 | FLAIR & T1WI & DWI |
| #34 | hemorrhage | 2 | 88.9 | 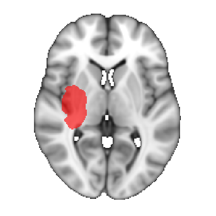 | FLAIR & T1WI & DWI |
| #35 | hemorrhage | 3 | 100.0 | 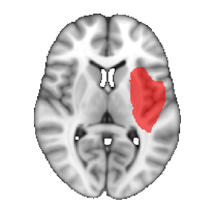 | FLAIR & T1WI & DWI |
| #36 | hemorrhage | 5 | 100.0 | 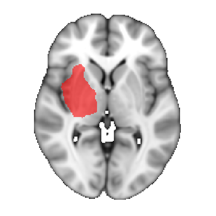 | FLAIR & T1WI & DWI |
| #37 | hemorrhage | 4 | 100.0 | 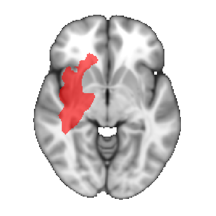 | FLAIR & T1WI & DWI |
| #38 | hemorrhage | 6 | 100.0 | 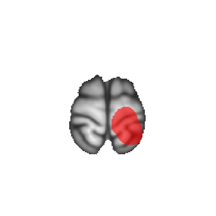 | FLAIR & T1WI & DWI |
| #39 | infarction | 82 | 88.9 | 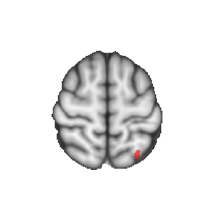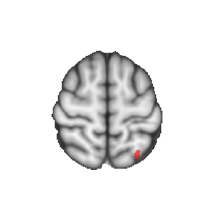 | T2WI |
| #40 | infarction | 8 | 55.6 | 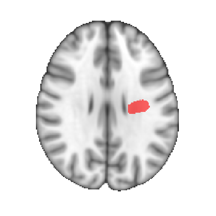 | FLAIR & DWI |
| #41 | infarction | 6 | 66.7 | 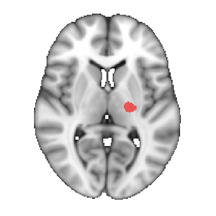 | FLAIR & DWI |
| #42 | infarction | 5 | 77.8 | 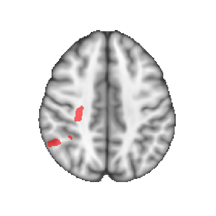 | T2WI & DWI |
| #43 | infarction | 2 | 77.8 | 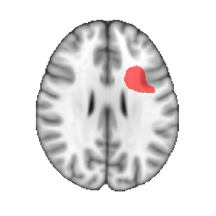 | FLAIR & DWI |
| #44 | infarction | 4 | 100.0 | 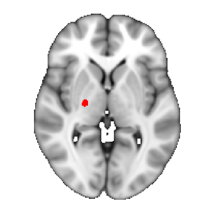 | FLAIR & DWI |
| #45 | infarction | 5 | 77.8 | 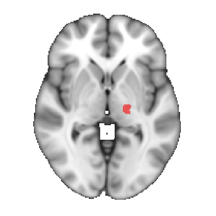 | FLAIR & DWI |
| #46 | infarction | 43 | 100.0 | 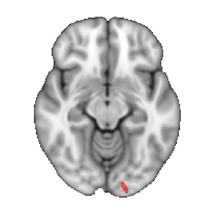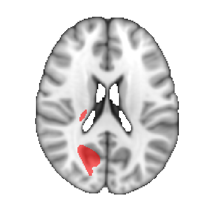 | T2WI & DWI |
| #47 | infarction | 8 | 100.0 | 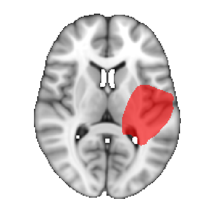 | T2WI & DWI |
| #48 | infarction | 5 | 100.0 | 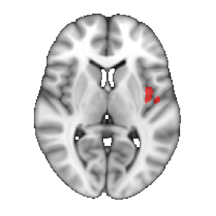 | FLAIR & DWI |
| #49 | infarction | 3 | 100.0 | 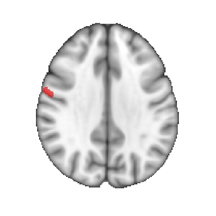 | FLAIR & DWI |
| #50 | infarction | 5 | 100.0 | 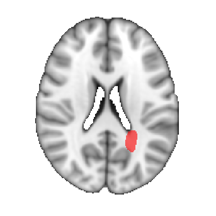 | FLAIR & DWI |
| #51 | infarction | 7 | 100.0 | 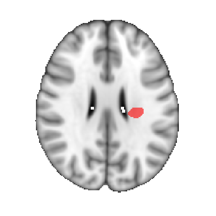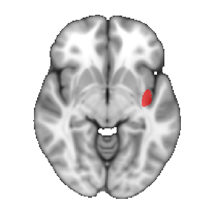 | T2WI & DWI |
| #52 | infarction | 8 | 100.0 | 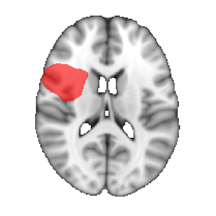 | FLAIR & DWI |
| #53 | infarction | 19 | 100.0 | 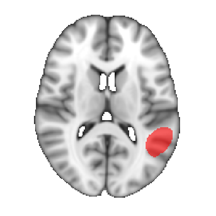 | T2WI & DWI |
| #54 | infarction | 2 | 88.9 | 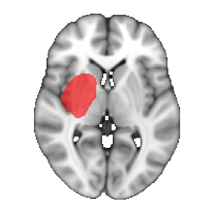 | T2WI & DWI |

Abbreviations: FLAIR, fluid-attenuated inversion recovery; DWI, diffusion-weighted imaging.

**Supplementary Table 2 - Clinical and demographic data of patients in the acute-to-subacute phase and the chronic phase**

| Characteristic | Acute to Subacute  (n = 18) | Chronic  (n = 10) | P value | Effect size |
| --- | --- | --- | --- | --- |
| Age (years) | 67.9 (14.8) | 72.6 (10.4) | 0.494 | r = 0.136 |
| Sex (M/F) | 13/5 | 6/4 | 0.507 | Φ = 0.125 |
| MMSE | 26.8 (3.9) | 26.2 (3.2) | 0.382 | r = 0.172 |
| SARA | 3.9 (4.8) | 9.5 (9.9) | 0.045 | r = 0.378 |
| Lesion volume (ml) | 18.5 (20.3) | 27.3 (27.9) | 0.524 | r = 0.127 |

Data are given as mean (SD), Abbreviations: MMSE, Mini-Mental State Examination; SARA, Scale for the Assessment and Rating of Ataxia.

**Supplementary Table 3 - Visual illusion task scores of patients in the acute-to-subacute phase and controls**

| Task | Cerebellar Stroke  (n = 18) | Non-Cerebellar  Stroke (n = 24) | Healthy Control  (n = 24) | F value  (df = 65) | P value | Effect size (η^2^) | Post hoc |
| --- | --- | --- | --- | --- | --- | --- | --- |
| Poggendorff task  (illusion rate %) | 76.5 (27.2) | 89.8 (13.1) | 89.8 (11.8) | 3.690 | 0.031 | 0.105 | HC, NCS > CS |
| Oblique control task  (correct rate %) | 94.4 (12.8) | 94.7 (12.5) | 94.4 (12.7) | 0.000 | 1.000 | 0.000 | - |
| Upper control task  (correct rate %) | 100 (0.0) | 98.7 (6.7) | 100 (0.0) | 0.872 | 0.423 | 0.027 | - |

Date are given as mean (SD), Abbreviations: df, degrees of freedom; HC, Healthy controls; CS, Cerebellar stroke; NCS, Non-cerebellar stroke

**Supplementary Table 4 - Correlation analysis between the illusion effect and each clinical characteristic**

|  | Cerebellar Strokes | |  | All Strokes | |
| --- | --- | --- | --- | --- | --- |
| Illusion rate vs. | Spearman r | p value |  | Spearman r | p value |
| Age (years) | -0.202 | 0.302 |  | -0.138 | 0.316 |
| Duration (days) | -0.171 | 0.385 |  | -0.185 | 0.176 |
| Acute to Subacute | -0.166 | 0.511 |  | -0.104 | 0.513 |
| MMSE | 0.050 | 0.799 |  | 0.109 | 0.430 |
| SARA | -0.158 | 0.423 |  | -0.158 | 0.423 |
| Lesion volume (ml) | -0.093 | 0.638 |  | -0.003 | 0.981 |

Abbreviations: MMSE, Mini-Mental State Examination; SARA, Scale for the assessment and rating of ataxia

**Supplementary Table 5 – Within-group analysis of the association between the illusion effect and each clinical characteristic**

|  |  | Cerebellar Strokes (n = 28) | | | |  | All Strokes (n = 55) | | | |
| --- | --- | --- | --- | --- | --- | --- | --- | --- | --- | --- |
|  |  | illusion rate | n | p value | effect size (r) |  | illusion rate | n | p value | effect size (r) |
| Sex | Male | 72.5 (26.5) | 19 | 0.575 | 0.106 |  | 81.7 (23.8) | 34 | 0.804 | 0.033 |
|  | Female | 79.0 (24.5) | 9 |  |  |  | 83.6 (18.1) | 21 |  |  |
| Etiology | Infarction | 76.9 (24.9) | 24 | 0.262 | 0.212 |  | 81.9 (22.0) | 40 | 0.849 | 0.0256 |
|  | Hemorrhage | 61.1 (29.4) | 4 |  |  |  | 83.7 (21.4) | 15 |  |  |
| Duration | Acute to Subacute | 76.5 (27.2) | 18 | 0.535 | 0.117 |  | 84.1 (21.1) | 42 | 0.293 | 0.142 |
|  | Chronic | 71.1 (23.5) | 10 |  |  |  | 82.4 (21.7) | 13 |  |  |

Data are given as mean (SD), All variables are assessed using the Mann–Whitney U test.

**Supplementary Figure 1. Poggendorff illusion rates of the patients in the acute-to-subacute phase and controls**

Average Poggendorff illusion rates are shown with error bars denoting ±1 standard error of the mean. The CS group shows a lower Poggendorff illusion rate than the NCS and HC groups (one-way ANOVA). Abbreviations: CS, Cerebellar stroke; NS, Non-cerebellar stroke, HC, Healthy controls. *p < 0.05.

**Supplementary Figure 2. Lesion overlap maps and SVR-LSM results of the patients in the acute-to-subacute phase**

A, Lesion overlap maps for patients in the acute-to-subacute phase. The color scale indicates the relative number of patients with overlapping lesions. B, Results of SVR-LSM in patients in the acute-to-subacute phase. Lesioned areas associated with significantly reduced Poggendorff illusion rates are shown in red to yellow. The color scale indicates the Z score. The results are thresholded at voxel-wise P < 0.005 and corrected for cluster size at P < 0.05 based on 10000 permutations. Data were corrected for lesion volume, sex, age, and disease duration, and the maximum voxel value was *Z* = 3.719 (MNI coordinates: 25, −59, −43).

**Supplementary Figure 3. Lesion overlap maps and VLSM results**

A, Lesion overlap maps for all patients. The color scale indicates the relative number of patients with overlapping lesions. B, Results of VLSM. Lesioned areas associated with significantly reduced Poggendorff illusion rates are shown in red to yellow. The color scale indicates the Z score. The threshold for statistical significance was *Z* = 3.485 (permutation FDR, p = 0.05), and the maximum voxel value was *Z* = 5.395 (MNI coordinates: 19, −56, −43).

**Supplementary Figure 4. Results of VLSM on the cerebellar flat map**

Lesioned areas associated with significantly reduced Poggendorff illusion rates are visualized on a cerebellar flat map using the SUIT toolbox for SPM. The color scale indicates the Z score. The threshold for statistical significance was *Z* = 3.485 (permutation FDR, p = 0.05), and the maximum voxel value was *Z* = 5.395.
