# Supplementary material for: Reduced likelihood of the Poggendorff illusion in cerebellar strokes: a clinical and neuroimaging study
Source: Brain Commun. 2023 Mar 6;5(2):fcad053. doi: 10.1093/braincomms/fcad053 (PMC10018644; doi:10.1093/braincomms/fcad053)
Supplement: fcad053_Supplementary_Data [file fcad053_supplementary_data.zip › Supplementary_material.docx]

**Materials and Methods of Voxel-Based Lesion-Symptom Mapping (VLSM)**

The normalized lesion map was analyzed using nonparametric mapping (NPM).^1^ First, lesion overlap was calculated to create a color-coded overlay map of lesion voxels across all stroke cases and to provide an overview of all lesion areas. Second, the statistical contribution of lesion location to the Poggendorff illusion rate was tested using VLSM. t-test statistics were used to compare the Poggendorff illusion rate on a voxel-by-voxel basis between dichotomous overlap values (lesion or no lesion in a given voxel) in the CS and NCS groups.^2^ Voxels that demonstrated lesions in ≥ 3% of the participants were analyzed. A 5% false discovery rate (FDR) correction with 3,000 permutations was used to correct for multiple comparisons. For all VLSM analyses, a minimum cluster size of 100 mm^3^ was employed.

**Reference**

1. Rorden C, Bonilha L, Fridriksson J, Bender B, Karnath HO. Age-specific CT and MRI templates for spatial normalization. *Neuroimage*. Jul 16 2012;61(4):957-65. doi:10.1016/j.neuroimage.2012.03.020

2. Rorden C, Karnath HO, Bonilha L. Improving lesion-symptom mapping. *J Cogn Neurosci*. Jul 2007;19(7):1081-8. doi:10.1162/jocn.2007.19.7.1081
